# Correlation between complement C3 level and abdominal aortic calcification in non-dialysis chronic kidney disease patients: a cross-sectional study

**DOI:** 10.3389/fmed.2026.1670422

**Published:** 2026-02-02

**Authors:** Chenfei Fu, Wen Li, Weijia Xu, Xinmei Huang, Wenqiong Cao, Aihua Zhang

**Affiliations:** 1 Department of Nephrology, Xuanwu Hospital, Capital Medical University, Beijing, China; 2 Department of Nephrology, Taihe Hospital, Hubei University of Medicine, Shiyan, Hubei, China; 3 Department of Nephrology, The First People’s Hospital of Lanzhou City, Lanzhou, Gansu, China; 4 National Clinical Research Center for Geriatric Disorders, Xuanwu Hospital, Capital Medical University, Beijing, China

**Keywords:** cardiovascular disease, chronic kidney disease, complement C3, non-dialysis, vascular calcification

## Abstract

**Background:**

Previous studies have shown that complement activation is associated with an increased risk of cardiovascular disease in patients with chronic kidney disease (CKD) on dialysis. This study aims to investigate the relationship between complement 3 (C3) and vascular calcification (VC) in non-dialysis-dependent CKD (NDD-CKD) patients.

**Methods:**

Four hundred and fifty six NDD-CKD patients were enrolled from 2019 to 2022. C3 was measured by immunoscattering turbidimetry. According to the results of CT imaging, the patients were divided into the aorta vascular calcification group and the non-aortic vascular calcification group. Restricted cubic spline (RCS) analysis and logistic regression model were used to explore the association between C3 and VC in NDD-CKD patients. To assess how it predicted, a receiver operating characteristic (ROC) analysis was undertaken.

**Results:**

In NDD-CKD patients, the third tertile of C3 (0.95–1.63 g/L) was significantly correlated with VC (OR = 2.06, 95% CI: 1.13–3.83, *p* = 0.020). Older age and history of underlying diseases (hypertension, hyperlipidemia) are independent risk factors for VC in NDD-CKD patients. The area under the curve (AUC) of C3 for predicting VC was 0.849 g/L (sensitivity, 79.4%; specificity, 76.7%).

**Conclusion:**

In patients with NDD-CKD, serum complement C3 exhibits a J-shaped relationship with vascular calcification, indicating a complex, non-linear association. Additionally, older age, hypertension, and hyperlipidemia were confirmed as independent risk factors.

## 1 Introduction

Cardiovascular events represent the primary determinant of poor prognosis in patients with CKD (1–4). VC, highly prevalent in CKD populations, strongly predicts cardiovascular mortality (5–7). Despite the established significance of VC, the clinical tools for its robust evaluation in routine practice remain suboptimal. Traditional biomarkers, such as serum calcium, phosphate, and parathyroid hormone (PTH), have been extensively studied. However, their predictive performance for VC has consistently been modest and inconsistent across studies. Moreover, other novel biomarkers, such as fibroblast growth factor-23 (FGF-23) and klotho, while mechanistically involved, have yet to demonstrate sufficient prognostic value or cost-effectiveness to be adopted into widespread clinical practice (8, 9). Recent studies have shown that complement activation is associated with many diseases and their complications (10). Nagaraj et al. (11) reported a significant correlation between higher C3 levels and the presence and severity of arterial calcification in healthy midlife women. Extending this concept to metabolic disease, Copenhaver et al. (12) demonstrated that elevated C3 and C4 levels in obese youth may serve as early indicators for atherosclerosis risk. Collectively, these findings position complement components as promising biomarkers for cardiovascular risk stratification (13).

This association has been confirmed in hemodialysis-dependent CKD (HD-CKD) patients, with C3 identified as an independent risk factor for VC (14). Nonetheless, the unique uremic milieu and intensive dialysis regimens in HD patients may confound the specific role of C3. Given the high incidence and progression of VC in NDD-CKD patients (15), reliance on HD data alone is insufficient for understanding this relationship at earlier, more modifiable disease stages. Thus, the utility of C3 as a VC biomarker in NDD-CKD patients remains an unaddressed gap. This study aims to explore the relationship between C3 and VC in NDD-CKD patients, providing novel insights for the targeted precision therapy of VC.

## 2 Materials and methods

### 2.1 Study subjects

**Figure 1:**
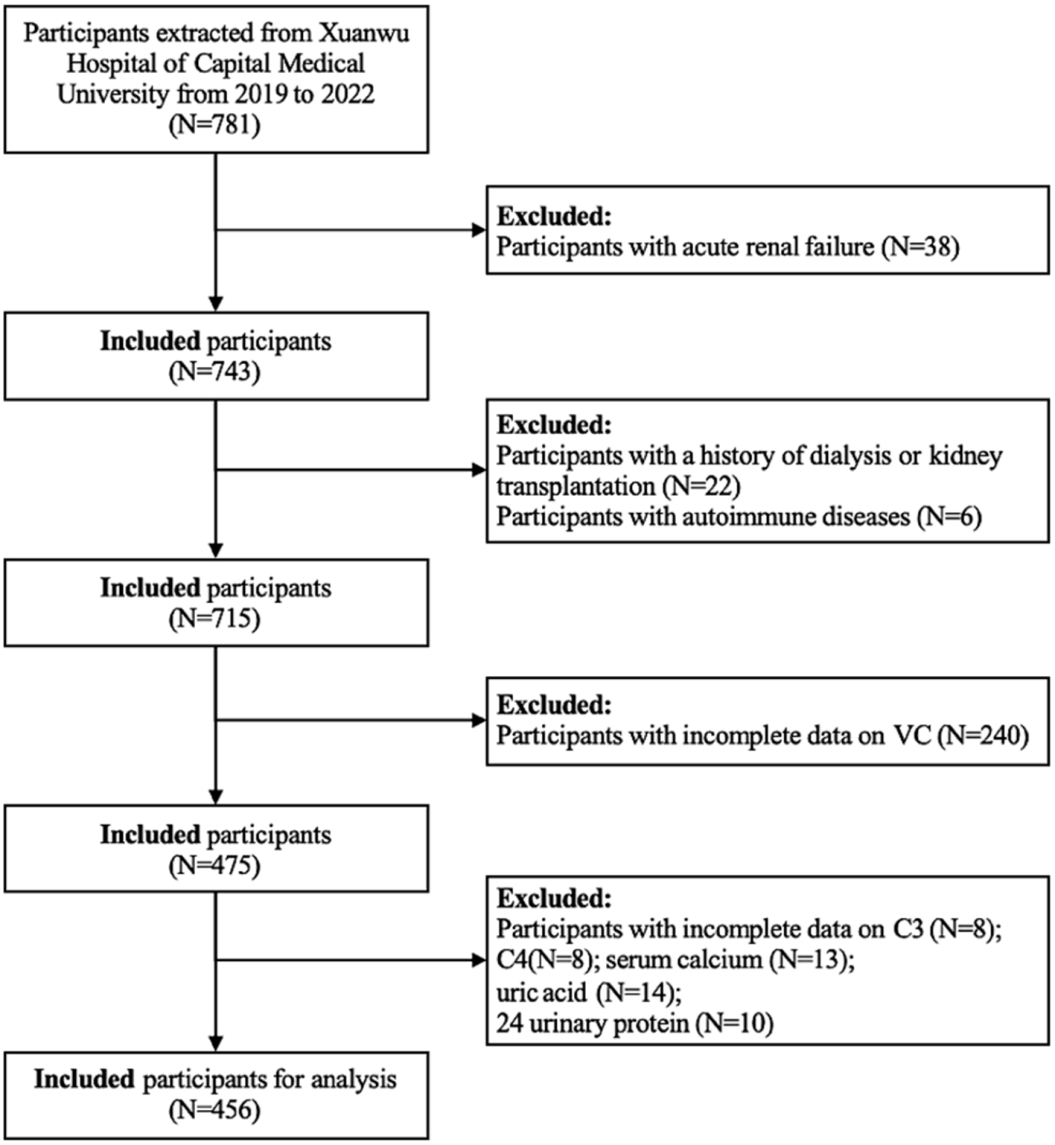
Flowchart of participants’ selection.

This cross-sectional observational study screened 737 NDD-CKD patients at Xuanwu Hospital of Capital Medical University from 2019 to 2022. Our exploration was restricted to subjects over 18 years old (*N* = 737). A series of sample selection processes were applied according to the exclusion criteria: (1) history of dialysis or kidney transplantation (*N* = 22); (2) patients with acute renal failure (*N* = 38); patients with active systemic inflammation or acute infection (*N* = 20); (3) autoimmune diseases (*N* = 6); (4) incomplete test for VC (*N* = 240); (5) missing data on available covariates: C3 (*N* = 8); C4 (*N* = 8); serum calcium (*N* = 13); uric acid (*N* = 253); 24 urinary protein (*N* = 46). Consequently, our study included 456 adult participants with complete information in the final analysis (Figure 1). The participants provided written informed consent before enrollment, and research protocols were approved by the Ethics Committee of Xuanwu Hospital, Capital Medical University [(2019)131].

### 2.2 Study methods

Clinical data were collected from 456 NDD-CKD patients, including demographics (gender, age, smoking status, alcohol use), etiology, CKD stage, blood pressure (systolic/diastolic), and medical history (hypertension, diabetes, chronic heart disease, hyperlipidemia, calcium carbonate/vitamin D supplementation). Serum levels of calcium, phosphorus, albumin, hemoglobin, uric acid, total cholesterol, low-density lipoprotein cholesterol (LDL-C), creatinine, and alanine were measured using an automatic biochemical analyzer. The levels of C3 and complement 4 (C4) were quantified via immunoscattering turbidimetry.

### 2.3 Assessment of vascular calcification

Vascular calcification (VC) was assessed using available chest CT imaging. The thoracic aorta was evaluated. VC was defined as discrete foci of ≥1 mm^2^ within the aortic wall with an attenuation of >130 Hounsfield units (HU), consistent with established methodologies (16).

## 3 Statistics

Data were analyzed using R (Version 4.3.2). Normally distributed continuous variables were described using mean ± standard deviation (SD) and compared via Student’s *t*-test. Non-normally distributed quantitative data were represented as median (interquartile range) [M (P25–P75)] and analyzed using the Mann–Whitney *U*-test. The categorical variables were described through absolute or relative frequencies (%), which were compared using the chi-square test (*p* < 0.05 considered significant). Spearman correlation analysis was used for correlation analysis. Multivariate logistic regression was performed to explore relationships between the indicators and VC in NDD-CKD patients. The predictive value of C3 concentration for VC was evaluated by the ROC curves. The nonlinear relationship between C3 and vascular calcification was assessed using restricted cubic splines.

## 4 Results

### 4.1 Comparison of clinical characteristics and laboratory indexes consisting of C3 and C4 levels in the group with and without VC

A total of 456 NDD-CKD patients were enrolled, including 233 with VC and 223 without VC. The general situation of the vascular calcification group and the non-vascular calcification group is presented in Table 1. The median age of the patients in the CKD with VC group was 65.00 (58.00–72.00) years. The median age of the patients in the CKD without VC group was 53.00 (42.00–64.00) years. By comparing patients with and without VC, the history of calcium carbonate or vitamin D supplementation showed no statistical difference (*p* > 0.05), whereas sex, smoking status, alcohol consumption, history of underlying diseases (hypertension, diabetes, chronic heart disease, hyperlipidemia), CKD stage, and cause of CKD were significantly associated with VC (*p* < 0.05).

**Table 1:** Comparison of baseline characteristics between those without VC and the VC groups in NDD-CKD patients.

Variables	CKD without VC group (*N* = 223)	CKD with VC group (*N* = 233)	*p*-value
Age [M (P25–P75), years]	53.00 (42.00–64.00)	65.00 (58.00–72.00)	<0.001^*^
Sex			0.007^*^
Male/[*n* (%)]	128 (57.4%)	164 (70.4%)	
Female/[*n* (%)]	95 (42.6%)	69 (29.6%)	
Smoking/[*n* (%)]	90 (40.4%)	132 (56.7%)	<0.001^*^
Drinking/[*n* (%)]	60 (26.9%)	98 (42.1%)	<0.001^*^
Underlying diseases			<0.001^*^
Hypertension/[*n* (%)]	166 (74.4%)	212 (90.9%)	
Diabetes/[*n* (%)]	80 (35.9%)	152 (65.2%)	
CHD/[*n* (%)]	28 (12.6%)	73 (31.3%)	
Hyperlipidemia/[*n* (%)]	112 (50.2%)	156 (67.0%)	
History of medications			
Calcium carbonate/[*n* (%)]	72 (32.3%)	60 (25.8%)	0.140
Vitamin D/[*n* (%)]	54 (24.2%)	44 (18.9%)	0.140
CKD stage			<0.001^*^
CKD1/[*n* (%)]	69 (30.9%)	25 (10.7%)	
CKD2/[*n* (%)]	34 (15.2%)	33 (14.2%)	
CKD3/[*n* (%)]	46 (20.6%)	82 (35.2%)	
CKD4/[*n* (%)]	36 (11.7%)	41 (17.6%)	
CKD5/[*n* (%)]	38 (17.0%)	52 (22.3%)	
Etiology of CKD			<0.001^*^
DKD/[*n* (%)]	41 (18.4%)	88 (37.8%)	
HRD/[*n* (%)]	13 (5.83%)	21 (9.01%)	
Glomerulonephritis/[*n* (%)]	169 (75.8%)	124 (53.2%)	
Serum phosphate (mean ± SD, mmol/L)	1.29 ± 0.40	1.34 ± 0.36	0.019^*^
Serum calcium (mean ± SD, mmol/L)	2.15 ± 0.21	2.16 ± 0.27	0.740
BMI (mean ± SD, kg/m^2^)	26.38 ± 4.44	26.16 ± 4.31	0.437
SBP (mean ± SD, mmHg)	142.04 ± 21.08	146.95 ± 21.17	0.011^*^
DBP (mean ± SD, mmHg)	86.57 ± 15.25	81.91 ± 11.93	<0.004^*^
24urinary protein (mean ± SD, g/24 h)	3.12 ± 2.73	2.95 ± 2.91	0.247
Creatinine (mean ± SD, μmol)	229.37 ± 243.92	263.06 ± 275.96	0.001^*^
Alanine (mean ± SD, IU/L)	22.18 ± 16.52	16.82 ± 9.95	<0.001^*^
Cholesterol (mean ± SD, mmol/L)	5.17 ± 2.07	4.64 ± 1.58	0.005^*^
Low-density lipoprotein (mean ± SD, mmol/L)	3.19 ± 1.72	2.79 ± 1.20	0.019^*^
ALB (mean ± SD, g/L)	33.77 ± 7.97	33.50 ± 6.63	0.318
C3 (mean ± SD, g/L)	0.91 ± 0.21	0.86 ± 0.20	0.011^*^
C4 (mean ± SD, g/L)	0.25 ± 0.08	0.25 ± 0.08	0.841
Uric acid [M (P25–P75), μmol/L]	401 (319–478.5)	414 (340–501)	0.062
Hemoglobin [M (P25–P75), g/L]	109 (94.5–133)	108 (93–119)	0.015^*^

Data are shown as mean ± SD or median (interquartile range) for continuous variables and proportions for categorical variables. *p* < 0.05 is marked with *.

VC, vascular calcification; CKD, chronic kidney disease; CHD, chronic heart disease; HRD, hypertensive renal damage; DKD, diabetic kidney disease; BMI, body mass index; SBP, systolic blood pressure; DBP, diastolic blood pressure; ALB, albumin; VC, vascular calcification; CKD, chronic kidney disease; C3, complement 3; C4, complement 4.

The VC group exhibited higher systolic blood pressure, creatinine, and serum phosphate than those in the group without VC (*p* < 0.05), and lower diastolic blood pressure, cholesterol, low-density lipoprotein cholesterol, and hemoglobin than those in the group without VC (*p* < 0.05). Serum calcium, body mass index (BMI), 24-h urinary protein, and uric acid did not differ between the two groups (*p* > 0.05).

When analyzed as continuous variables, C3 showed a significant negative association with VC (*p* < 0.05), with a monotonic downward trend in the non-VC group, while C4 was not statistically significant.

### 4.2 Correlation between C3 and VC and laboratory indicators

**Figure 2:**
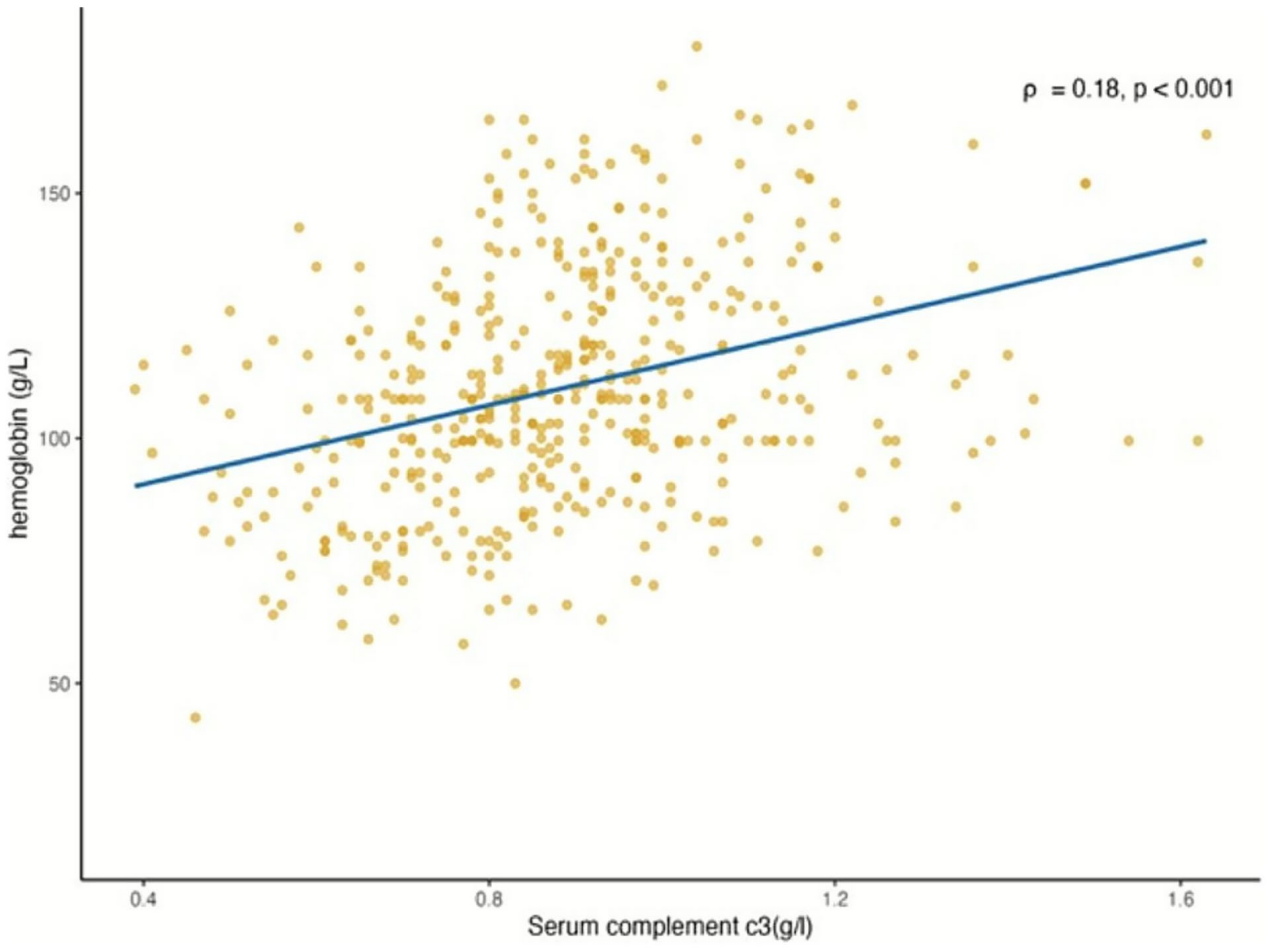
Correlation between plasma C3 and hemoglobin in NDD-CKD patients. NDD-CKD, non-dialysis chronic kidney disease. Correlation between plasma C3 level and hemoglobin in NDD-CKD patients, Spearman’s *ρ* = 0.18, *p* < 0.001.

Initial Spearman correlation analysis showed that C3 was negatively correlated with VC (*r* = −0.113, *p* = 0.015) and positively correlated with hemoglobin, alanine, cholesterol, and low-density lipoprotein cholesterol (*ρ* = 0.352, 0.264, 0.183, 0.163, *p* < 0.001) (see Figure 2).

### 4.3 Nonlinear association between C3 and traditional VC risk factors

**Figure 3:**
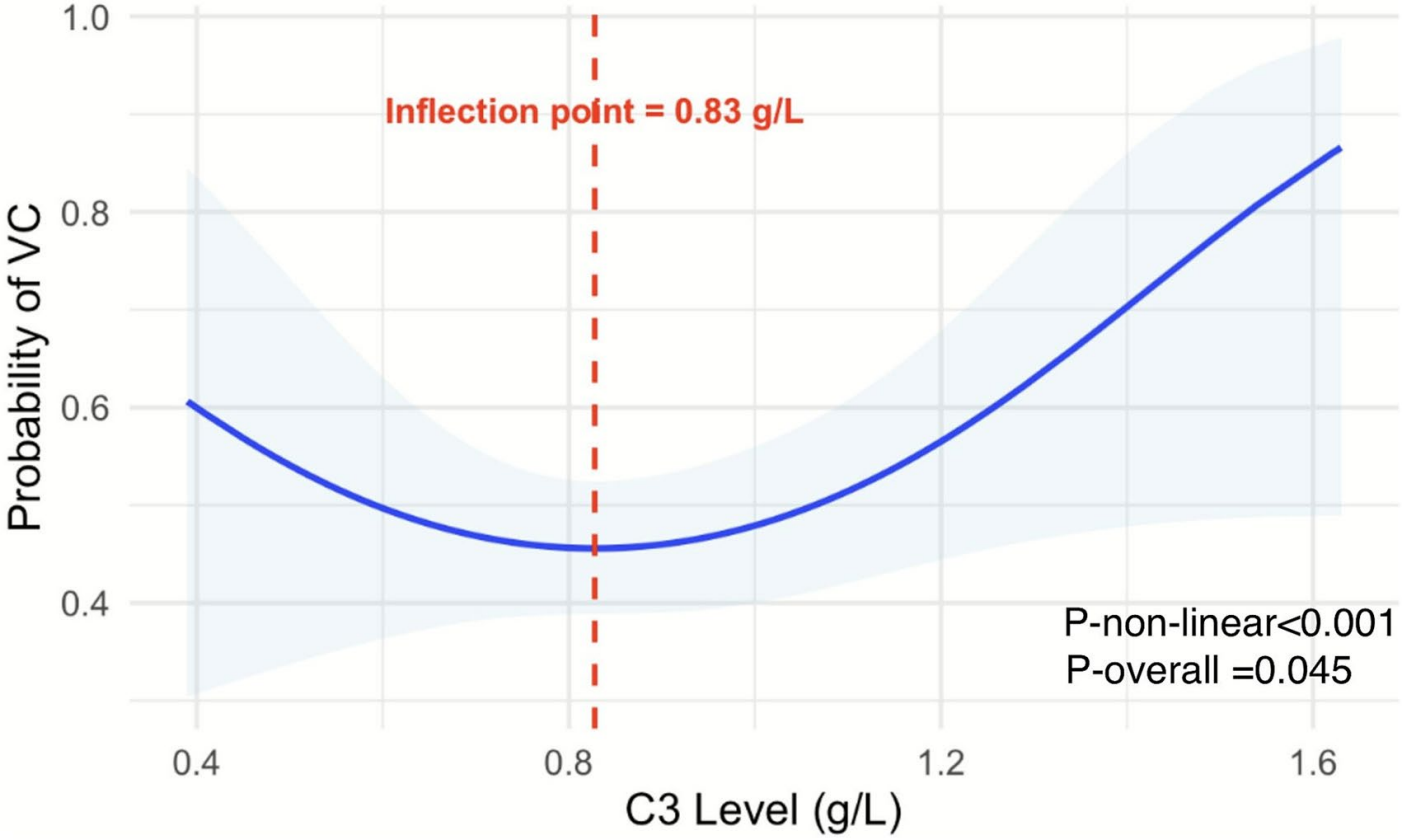
Nonlinear association between C3 and vascular calcification. VC, vascular calcification. Restricted cubic spline analysis revealed a significant non-linear J-shaped association between C3 levels and vascular calcification risk (*p*-non-linear <0.001), with overall statistical significance (*p*-overall = 0.045).

Given the complex biology of the complement system, we further used restricted cubic spline (RCS) curve analysis to explore possible nonlinear relationships between C3 and VC. The RCS analysis confirmed a J-shaped relationship between C3 and VC (*p* = 0.045), with an inflection point at 0.83 g/L. Below or above this value, the risk of VC increased (Figure 3).

### 4.4 Analysis of influencing factors of VC in NDD-CKD patients

The J-shaped relationship clarifies that the risk of VC is not uniformly related to C3 across its entire range, and the mean value may mask the true relationship. To further investigate this, C3 was divided into three subgroups by tertiles: C3_quartile.L (0.39–0.80 g/L), C3_quartile.Q (0.81–0.94 g/L), and C3_quartile.C (0.95–1.63 g/L). The C3_quartile.Q was set as the baseline because it encompasses the inflection point (0.83 g/L) and thus represents the patient group with the lowest risk.

**Figure 4:**
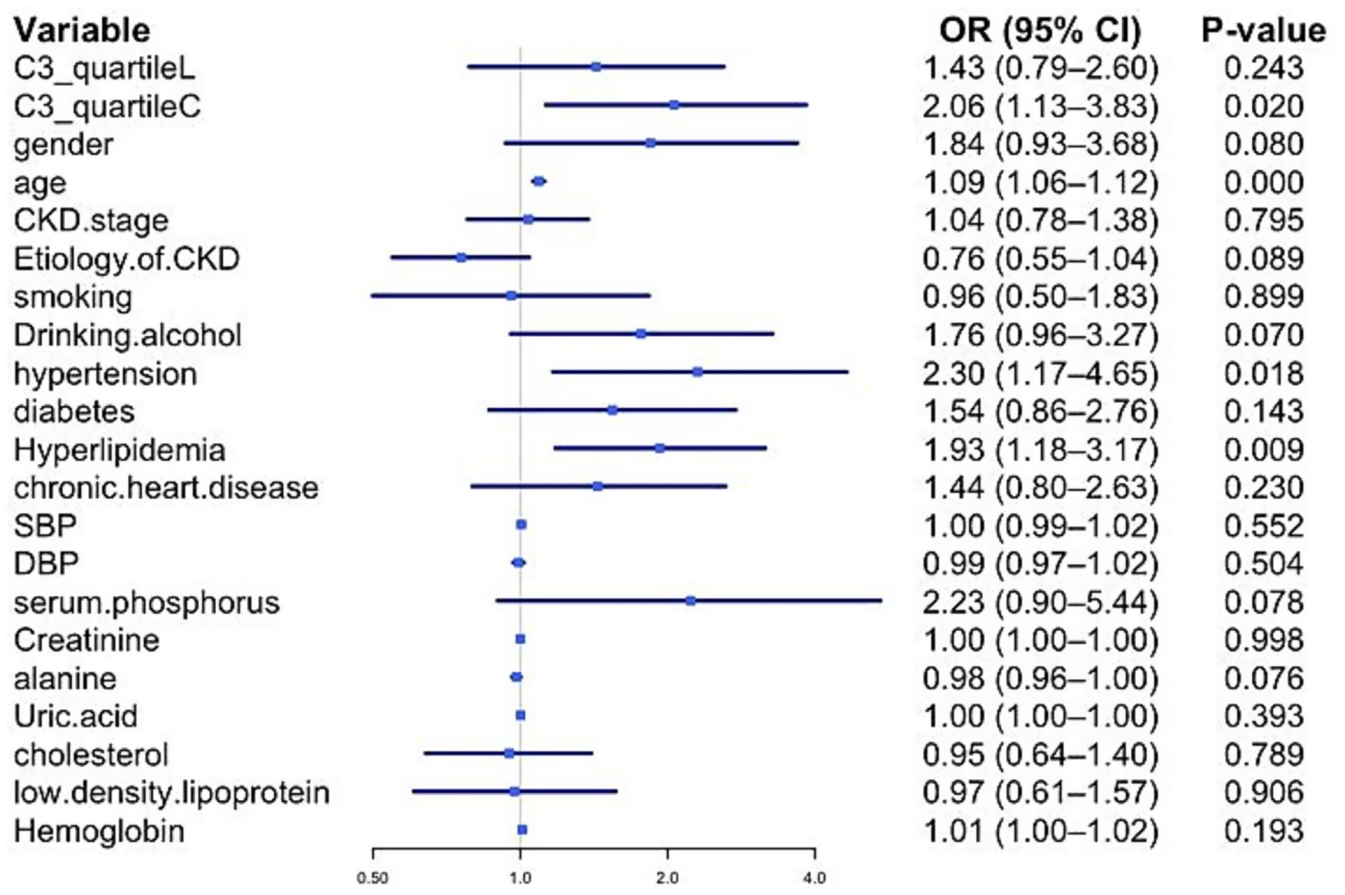
Estimated odds ratios determined in a logistic regression model. OR, odds ratio; CI, confidence interval. Among 17 risk factors, only C3_quartileC, age, hypertension, and hyperlipidemia prevalence had a *p*-value <0.05.

Seventeen parameters with *p* < 0.1 in univariate logistic regression were included in multivariate analysis (Figure 4). The high C3 tertile (C) was significantly associated with VC [odds ratio (OR) = 2.06, 95% confidence interval (CI): 1.13–3.83, *p* = 0.020], whereas the low tertile (L) showed no significant association. Despite a lower mean C3 in the VC group, the highest C3 tertile conferred a twofold VC risk (OR = 2.06). This apparent contradiction can be explained by the J-shaped relationship identified in the RCS analysis. Despite a lower mean C3 in the VC group, the highest C3 tertile conferred a twofold VC risk (OR = 2.06). This apparent contradiction can be explained by the J-shaped relationship identified in the RCS analysis. The J-shaped curve indicates that both low and high levels of C3 are associated with an increased risk of VC, with the lowest risk occurring at an intermediate C3 level (0.81–0.94 g/L). Therefore, while the mean C3 level in the VC group is lower, the highest C3 tertile still represents a significant risk factor for VC.

In addition to C3, older age, hypertension, and hyperlipidemia were identified as independent risk factors for VC in NDD-CKD patients.

### 4.5 The predictive value of C3 for VC in NDD-CKD patients

The ROC curve was used to analyze the ability of C3 to diagnose vascular calcification, which showed an area under the curve (AUC) of 0.849 (95% confidence interval: 0.814–0.884) for C3 in predicting VC, indicating high diagnostic accuracy (Table 2).

**Table 2:** The predictive value of C3 for VC in NDD-CKD patients.

Cut-off	Sensitivity	Specificity	Accuracy	AUC	AUC 95% CI
0.534	0.794	0.767	0.781	0.849	0.849 (0.814–0.884)

AUC, area under the curve; CI, confidence interval; C3, complement3; VC, vascular calcification; NDD-CKD, non-dialysis dependent CKD.

### 4.6 Subgroup analysis by glomerulonephritis status

Given the distinct role of the complement system in GN pathophysiology, we conducted subgroup analyses to examine how the presence or absence of GN status affected the relationship between C3 and vascular calcification. We observed a significant positive association in patients with GN (OR = 6.01, *p* = 0.033). In contrast, the association in the non-GN subgroup did not reach statistical significance (OR = 0.018, *p* = 0.066).

## 5 Discussion

This study provides the first exploration of the association between C3 and VC in patients with NDD-CKD. Our findings reveal a significant J-shaped relationship (*p* = 0.045), with an inflection point at 0.83 g/L. This indicates that both low and high C3 levels are associated with an increased risk of VC, revealing a previously unrecognized complexity in their association.

Previous studies have demonstrated that the complement system may play a crucial role in the development of VC (17). A study in subclinical atherosclerosis has identified complement system activation as a hallmark of early plaque formation (18). Complement C3 is the core of the complement system, with the highest serum content in all complement components, and is the intersection hub of complement activation pathways (19). In hemodialysis patients, Wang et al. (14) have demonstrated that C3 acts as a predictor of vascular calcification. Our study also confirmed the association of C3 and vascular calcification in non-hemodialysis patients. However, unlike the linear relationship between C3 and VC in hemodialysis patients (14), a J-shaped relationship was observed in NDD-CKD patients in our study. This difference may be attributed to complement activation’s involvement in hypersensitivity reactions (such as CARPA) and acute reactions during HD. The uremic environment and recurrent inflammatory stimuli during dialysis may significantly alter C3 metabolism compared to the non-dialysis state (20).

The J-shaped relationship demonstrates that the apparent paradox, a lower mean C3 level in the VC group alongside an elevated risk in the highest C3 tertile, arises because the population mean obscures a high-risk subgroup driven by elevated C3 levels. Similar to bone formation, vascular calcification is a process of ectopic bone formation influenced by factors like abnormal mineral metabolism, uremic toxins, inflammation, and oxidative stress (21, 22). On one hand, complement C3 has a dual role in inducing osteoclast formation (23) and regulating the inflammatory environment, impacting osteoclast differentiation (24). This enhanced osteoclast activity can lead to bone loss, increasing blood calcium levels, and the risk of soft tissue calcification in vascular walls, which may promote vascular calcification (25, 26). On the other hand, our study showed that low complement C3 levels also caused vascular calcification. The complement system is not only involved in the inflammatory response, but also in the removal of cellular debris and apoptotic cells (10). We propose that low C3 levels may result in impaired clearance of these functions, allowing cell debris and apoptotic cells to accumulate in the vessel wall. This accumulated cellular debris can act as an inducer of calcification and promote the formation of hydroxyapatite crystals, which can lead to vascular calcification (27). In addition to low C3 levels, an imbalance in complement regulation, potentially caused by excessive activation, could be a significant factor. This idea is supported by the predictive value of the C3d/C3 ratio, which reflects the balance between activation and abundance (28). Studies have shown that deficiencies in regulators like CD59 can worsen vascular disease (29). To better understand the role of complement dysregulation in vascular calcification, future research should focus on quantifying complement activation fragments and regulators directly. While our study highlights the importance of complement dysregulation, it is essential to recognize that vascular calcification is a complex process involving multiple factors, including the FGF-23–Klotho axis and oxidative stress (30–32). Future prospective studies should measure these factors in conjunction with complement components to better understand their interactions and contributions to VC.

Clinically, NDD-CKD patients with VC were significantly older than patients without VC (65 years vs. 53 years), had higher systolic blood pressure, lower diastolic blood pressure, cholesterol, low-density lipoprotein, and hemoglobin, and higher rates of hypertension, diabetes, and hyperlipidemia prevalence (all *p* < 0.05), showing no difference in calcium/vitamin D supplementation. This is generally consistent with prior studies (33–35). Meanwhile, in this study, C3 was found to be positively correlated with hemoglobin, total cholesterol, and LDL-C, markers of inflammatory responses and metabolic disorders (36). Inflammation and metabolic disorders are important risk factors for VC, suggesting indirect VC promotion via these pathways, while this study did not demonstrate a significant relationship between C4 and VC, aligning with studies in hemodialysis patients. However, Bi et al. (37) identified C4 as a prognostic marker in IgA nephropathy patients. This study did not include prognostic analysis due to the short follow-up time, but future studies should explore the association of plasma C4 levels with cardiovascular disease (CVD) and mortality in NDD-CKD patients.

The impact of C3 activation on vascular calcification (VC) may vary depending on the underlying etiology of kidney disease, as complement dysregulation is a common feature in various glomerulonephritides, including IgA nephropathy, lupus nephritis, APS, and others, as documented in previous studies (38, 39). This diversity in etiology prompted us to stratify our analysis by glomerulonephritis (GN) status. Our analysis demonstrated a significant association between higher C3 levels and an increased risk of VC in the GN subgroup. However, in the non-GN subgroup, this association did not reach statistical significance, likely due to the limited statistical power of the smaller cohort (approximately 100 patients). Future large-scale, multicenter studies with etiology-specific designs are needed to validate these potential differences and elucidate the underlying pathophysiology.

The ROC curve showed that the AUC of C3 in predicting VC in NDD-CKD patients was 0.849 g/L, indicating a high predictive value. The predictive sensitivity and specificity were 79.4 and 76.7%, respectively. C3 may be an important biomarker for predicting VC in NDD-CKD patients.

In clinical practice, C3 serves as a valuable biomarker for assessing the risk of vascular calcification in patients with NDD-CKD. In patients with markedly altered C3 levels, whether elevated or decreased, especially among the elderly with hypertension and hyperlipidemia, close monitoring for signs of VC (using methods such as X-ray) is essential for the early identification of high-risk individuals. In addition to monitoring, the trend in C3 levels can guide clinical management decisions. Elevated C3 levels, particularly those in the high-normal range, may require closer monitoring, whereas low C3 levels may indicate complement dysregulation or associated comorbidities. This targeted approach facilitates more precise interventions, ultimately improving patient outcomes.

This study has several limitations that should be considered when interpreting the findings. First, we could only classify vascular calcification as present or absent due to the lack of a standardized grading system for aortic calcification on CT scans. While our method was reliable, it may not fully capture the range of disease severity. Second, a single cross-sectional C3 measurement may not accurately reflect long-term complement dynamics, though we mitigated this by excluding patients with acute comorbidities known to cause transient fluctuations. Additionally, medication use was documented from medical records, but real-world adherence may not have been fully captured. Finally, as a single-center study, our findings require validation in more diverse populations. Despite these limitations, the consistency and robustness of the primary nonlinear association provide compelling evidence for a complex relationship between C3 and vascular calcification, laying the groundwork for future longitudinal studies with quantitative calcification scoring.

Collectively, these findings deepen our understanding of the possible mechanisms of VC. With the ongoing development of C3-targeted therapies, in the future, C3 may be applied to the clinical treatment of VC in CKD, thereby improving the quality of life for patients and achieving better survival benefits.

## 6 Conclusion

In patients with NDD-CKD, serum complement C3 exhibits a J-shaped relationship with vascular calcification, indicating a complex, non-linear association. Additionally, older age, hypertension, and hyperlipidemia were confirmed as independent risk factors.

## Data Availability

The raw data supporting the conclusions of this article will be made available by the authors, without undue reservation.
